# Associations of plasma phosphorylated tau181 and neurofilament light chain with brain amyloid burden and cognition in objectively defined subtle cognitive decline patients

**DOI:** 10.1111/cns.13962

**Published:** 2022-09-08

**Authors:** Yanlu Huang, Yuehua Li, Fang Xie, Qihao Guo

**Affiliations:** ^1^ Department of Gerontology Shanghai Jiao Tong University Affiliated Sixth People's Hospital Shanghai China; ^2^ Department of Radiology Shanghai Jiao Tong University Affiliated Sixth People's Hospital Shanghai China; ^3^ PET Center, Huashan Hospital Fudan University Shanghai China

**Keywords:** neurofilament light chain, objectively defined subtle cognitive decline, phosphorylated tau181, plasma biomarkers

## Abstract

**Aims:**

There is increasing evidence that plasma biomarkers are specific biomarkers for Alzheimer's disease (AD) pathology, but their potential utility in Obj‐SCD (objectively defined subtle cognitive decline) remains unclear.

**Methods:**

A total of 234 subjects, including 65 with brain amyloid beta (Aβ) negative normal cognition (Aβ− NC), 58 with Aβ‐positive NC (Aβ+ NC), 63 with Aβ− Obj‐SCD, and 48 with Aβ+ Obj‐SCD were enrolled. Plasma Aβ42, Aβ40, Aβ42/Aβ40 ratio, phosphorylated tau181 (p‐tau181), neurofilament light chain (NfL), and total tau (T‐tau) were measured using Simoa assays. Logistic and linear regression analyses were used to examine the relationship between plasma biomarkers and brain amyloid, cognition, and imaging measures adjusting for age, sex, education, APOE ε4 status, and vascular risk scores. Receiver operating characteristics were used to evaluate the discriminative validity of biomarkers.

**Results:**

After adjustment, only plasma p‐tau181 and NfL were significantly elevated in Aβ+ Obj‐SCD participants compared to Aβ− NC group. Elevated p‐tau181 was associated with brain amyloid accumulation, worse cognitive performance (visual episodic memory, executive function, and visuospatial function), and hippocampal atrophy. These associations mainly occurred in Aβ+ individuals. In contrast, higher NfL was correlated with brain amyloid burden and verbal memory decline. These associations predominantly occurred in Aβ− individuals. The adjusted diagnostic model combining p‐tau181 and NfL levels showed the best performance in identifying Aβ+ Obj‐SCD from Aβ− NC [area under the curve (AUC) = 0.814], which did not differ from the adjusted p‐tau181 model (AUC = 0.763).

**Conclusions:**

Our findings highlight that plasma p‐tau181, alone or combined with NfL, contributes to identifying high‐risk AD populations.

## Graphical abstract

This study provides strong support for using plasma biomarkers to identify potential risks for early Alzheimer's disease (AD). The figure presents the combination of plasma p‐tau181 and NfL can effectively identify AD pathology Obj‐SCD (objectively defined subtle cognitive decline).

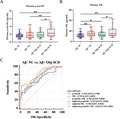

## 1 INTRODUCTION

As the development of new anti‐amyloid therapies moves toward the earliest clinical and preclinical stages of Alzheimer's disease (AD), there is an urgent need for accessible and reliable biomarkers to identify high‐risk populations for AD clinical trials. The typical pathophysiological features of AD are amyloid beta (Aβ), tau, and neurodegeneration.^1^ Due to the pathological heterogeneity and clinical presentation diversity of AD, clinical assessment can only improve the accuracy of AD diagnosis when combined with neuropathological biomarkers. Currently, amyloid‐Positron Emission Tomography (PET), tau‐PET, and cerebrospinal fluid (CSF) are the gold standard for detecting amyloid and tau pathology in clinical trials.^1^, ^2^ However, PET scans are expensive and still not widely available, whereas CSF sampling through lumbar punctures is invasive and unappealing. They have limited availability, especially in primary care. Therefore, there is a great need for less intrusive, cost‐effective, and easily available biomarkers, preferably blood tests. The rapid development of ultrasensitive assays makes it possible to predict the levels of proteins associated with pathological AD in blood. Some plasma biomarkers have been shown to correlate closely with CSF biomarkers and imaging markers.^3^, ^4^ The core blood biomarkers of AD include Aβ42 (or Aβ42/40 ratio), phosphorylated tau181 (p‐tau181), neurofilament light chain (NfL), and total tau (T‐tau), which reflect Aβ pathology, tau pathology, and neuronal damage, respectively.^5^, ^6^

Various experimental results have shown that lower plasma Aβ42/Aβ40 ratio was associated with higher amyloid cortical burden,^7^, ^8^ faster Aβ accumulation rates,^9^ greater cognitive declines,^10^, ^11^ and increased risk of developing AD dementia at follow‐up.^12^ Other research suggested plasma p‐tau181 reflected changes in hyperphosphorylated tau in the brain in Aβ+ individuals.^13^, ^14^ Cross‐sectional and longitudinal studies reported plasma p‐tau181 was associated with more significant memory decline, the rate of clinical progression to dementia, hypometabolism, cortical thinning, and gray matter volume atrophy in highly AD‐characteristic brain regions.^13^, ^15^, ^16^, ^17^ Except that plasma p‐tau181 was proved to have an excellent diagnostic power in distinguishing AD dementia from Aβ− negative individuals and other neurodegenerative diseases [AUC (area under the curve) >90%].^18^, ^19^ Baseline NfL was found related to the rate of Aβ deposition at 2‐year follow‐up in the left‐posterior cingulate and the inferior parietal gyri.^20^ A large cohort found that plasma NfL levels were increased in AD and correlated with cerebral metabolic deficits, brain atrophy, and cognitive decline.^21^, ^22^ Studies on plasma T‐tau had shown that it was not significantly related to the risk of dementia.^23^, ^24^ However, higher plasma T‐tau levels were associated with a significant decrease in cognitive performance, brain atrophy, and hypometabolism.^24^, ^25^, ^26^

Most studies focus on the relationships between plasma biomarkers and brain Aβ or cognition in participants with normal cognition (NC), mild cognitive impairment (MCI), or AD. However, few researchers explored the change and value of plasma biomarkers in the pre‐MCI stage. Obj‐SCD (objectively defined subtle cognitive decline) is a phase that shows very minimal cognitive changes but is not sufficient to warrant a diagnosis of MCI.^1^ Previous work has demonstrated that Obj‐SCD status predicts progression of MCI and dementia, accumulation of amyloid, the decline in daily function, cortical thinning, and alterations in cerebral blood flow.^27^, ^28^, ^29^ Recently, individuals with Obj‐SCD showed elevated baseline plasma NfL relative to the cognitively normal group, and higher NfL levels predicted a faster rate of decline in memory and composite score in Obj‐SCD.^22^ Another study found that Obj‐SCD/p‐tau181‐positive participants had rapid rates of amyloid accumulation, cognitive decline, and functional decline.^17^ However, the performance of other plasma biomarkers (Aβ42, Aβ40, Aβ42/Aβ40 ratio, and T‐tau) in Obj‐SCD and the relationship between plasma biomarkers and the brain Aβ, different cognition domains, and image measures are unclear.

Therefore, the purpose of this study is to examine the performance of plasma biomarkers, including Aβ42, Aβ40, Aβ42/Aβ40 ratio, p‐tau181, NfL, and T‐tau in Obj‐SCD relative to NC cross‐sectionally and whether baseline plasma biomarkers affect brain Aβ, cognition domains, and image indicators. In addition, we also attempted to explore the discriminatory value of plasma biomarkers in identifying Obj‐SCD from NC.

## 2 MATERIALS AND METHODS

### 2.1 Participants

In the study, 234 participants, including 123 NC and 111 Obj‐SCD participants, from the Shanghai community through advertisements from August 2019 to December 2021. All procedures were performed with the approval of the Ethics Committee of Shanghai Jiao Tong University Affiliated Sixth People's Hospital. We confirm that all methods were performed according to the relevant guidelines and regulations. Before study participation, participants provided written informed consent for study participation.

Participants who met the following criteria were included: (a) over 50 years old; (b) having at least 6 years of formal education; (c) having normal vision and hearing; (d) the score of MMSE (Chinese Version Of The Mini‐Mental State Examination)^30^ ≥24; (e) No history of severe head trauma or neurologic disease; and (f) can complete the examination of neuropsychological tests, MRI scan, amyloid PET, and blood tests.

Inclusion criteria of the NC group: (a) no memory complaints or memory loss observed; (b) normal performance on neuropsychological assessment [within 1 SD (standard deviation) of the average for age, gender, and education]; (c) the score of HAMD (Hamilton Depression Rating Scale)^31^ over the past 2 weeks ≤12.

Inclusion criteria of the Obj‐SCD group: (a) not meet Jak/Bondi criteria for MCI;^32^ and (b) had and only had one indicator of impairment (>1 SD below the demographically adjusted mean) in two different cognitive domains (memory, language, and executive function).

### 2.2 Neuropsychological measures

The neuropsychological battery included eight tests covering seven domains: Global cognition: (MMSE); Verbal episodic memory: immediate recall (IR) and long delay recall (LDR) from Auditory Verbal Learning Test (AVLT);^33^ Visual episodic memory: IR and LDR from Brief Visuospatial Memory Test‐Revised (BVMT‐R);^34^ Language function: Animal Verbal Fluency Test (AFT),^35^ and Boston Naming Test (BNT);^36^ Attention: forward score and backward score of Digit Span Test (DST);^37^ Executive function: Shape Trail Test (STT) A and STT B completion times;^38^ Visuospatial function: Judgment of Line Orientation (JLO).^39^ Higher scores for executive function signified worse function, while the higher scores signified better cognitive functioning for the rest of the assessments. Based on the domain groupings above, these test‐specific z scores were averaged to obtain a domain‐specific z score.

### 2.3 Composite vascular risk scores

Medical records or self‐reports established vascular risk factors during a medical history interview. Composite vascular risk scores were calculated for five vascular risk factors: hypertension, hyperlipidemia, diabetes, current smoking (within the past month), and obesity based on body mass index >30 kg/m^2^. Each risk factor was coded as 1/0 scores according to the presence or absence. In addition, the composite vascular risk scores were dichotomously coded according to the presence or absence of vascular risk factors.

### 2.4 Florbetapir PET

PET imaging using the 18F‐florbetapir AV‐45 tracer was used to quantify amyloid burden. Amyloid PET images were obtained from a PET/CT system (Biograph mCT Flow PET/CT, Siemens, Erlangen, Germany) at the PET center of Huashan Hospital, Fudan University. Briefly, PET images were firstly coregistered to individual T1‐weighted MR images. Transformative parameters from normalizing the MR images were used to warp the images into MNI space. Finally, a Gaussian kernel of [8 8 8] in full width at half maximum (FWHM) was used for smoothing the PET data. The global cortical Aβ accumulation standard uptake value ratio (SUVR) was obtained using the whole cerebellum as the reference region. The Positive 18F‐florbetapir PET images were independently judged by three physicians based on the guidelines for interpreting amyloid PET.^40^

### 2.5 Structural MRI

The details of MRI data acquisition and processing had been described in a previous study.^41^ The FreeSurfer image analysis software package (version 6.0) was used to process cortical thickness and hippocampal volume. Mean cortical thickness was averaged by the left and right cortical thickness. Relative hippocampal volumes were calculated by dividing whole hippocampal volumes by estimated total intracranial volume (eTIV) and multiplying by 1000.

### 2.6 Plasma biomarkers

Centrifuged plasma was aliquoted into ultra‐low adsorption tubes (AXYGEN MCT‐150‐L‐C) and stored at a −80°C refrigerator. The measurements of plasma biomarkers were performed on the Quanterix Simoa HD‐1 platform^42^ with Neurology 3‐Plex A Assay Kit (Lot 502,838). All analytical procedures were performed by laboratory technicians who maintained the confidentiality of clinical data in accordance with manufacturing instructions.

### 2.7 APOE genotyping

Following the manufacturer's protocol, the genomic DNA was extracted from blood samples with the Spin Columns DNA Isolation Kit (Generay Biotech Co., Ltd, Shanghai, CN). In accordance with the manufacturer's instructions, two polymorphic sites of APOE, rs7412 and rs429358, were detected by a ligase detection reaction (LDR) fluorescent nanosphere technique (NEB Company, United States).

### 2.8 Statistical analysis

Statistical analyses were performed using IBM SPSS Statistics for Windows (version 23.0). Pearson chi‐square tests were performed for categorical variables. We used the Shapiro–Wilk test to test data for normal distribution. One‐way analyses of variance (ANOVA) were performed for normally distributed continuous data. The Kruskal–Wallis test was used when the variances were not homogeneous. Pearson's correlation and *t*‐tests were used for the univariate analysis of variables (age, sex, education, APOE ε4 status, vascular risk scores) potentially associated with plasma biomarkers. Analysis of covariance (ANCOVA) was used to assess the relationship between diagnosis and plasma biomarkers, adjusting for age, sex, education, APOE ε4 status, and vascular risk scores. Bonferroni corrected post hoc tests were performed for group comparisons. Binary logistic regression was performed to assess each plasma biomarker's relationships and brain Aβ status (categorical variable). Linear regression was performed to examine the relationships between each plasma biomarker and brain Aβ SUVR (continuous variable), cognition domains, and imaging measures adjusted for the above covariates. All plasma biomarkers in the regression analysis were standardized to z scores to facilitate comparisons between models. Discrimination accuracies of biomarkers were determined with receiver operating characteristic (ROC) curve analysis by MedCalc 19.5.6.

## 3 RESULTS

### 3.1 Demographic characteristics

The detailed demographic, clinical characteristics, neuropsychological measures, and imaging data by groups of participants are shown in Table 1. The study included 234 individuals (66.2% females), with 65 in the Aβ−PET negative (Aβ−) NC group, 58 in the Aβ−PET positive (Aβ+) NC group, 63 in Aβ− Obj‐SCD group, and 48 in the Aβ+ Obj‐SCD group. The mean age was 64.25 ± 6.86 years, and the average length of their education was 12.32 ± 2.97 years. There were no significant differences between the four groups in demographic characteristics, except the SUVR was significantly higher in the Aβ+ group than in the Aβ− group (*p* < 0.001). In neuropsychological tests, Obj‐SCD participants showed poorer performance in global cognition, verbal episodic memory, visual episodic memory, language function, executive function, and attention than in the NC group (*p* < 0.014). In imaging measures, the Aβ+ obj‐SCD group showed significantly lower relative hippocampus volume than Aβ− NC (*p* = 0.010). No significant differences were observed between the same clinical diagnostic groups in neuropsychological tests and imaging measures.

**TABLE 1:** Characteristics of all participants

Characteristic	Aβ− NC *n* = 65	Aβ+ NC *n* = 58	Aβ− Obj‐SCD *n* = 63	Aβ+ Obj‐SCD *n* = 48	Test statistics	*p* Value
Age (years) (range 50–80)	62.98 ± 6.56	65.05 ± 6.77	64.51 ± 6.88	64.67 ± 7.31	1.091	0.354
Education (years) (range 6–20)	12.75 ± 2.89	12.54 ± 3.01	12.21 ± 2.98	11.62 ± 2.97	1.493	0.217
Sex (% Female)	48 (73.8)	32 (55.2)	41 (65.1)	34 (70.8)	5.349	0.148
APOE (% ε4)	15 (23.1)	14 (24.1)	19 (30.2)	14 (29.2)	1.166	0.761
SUVR (range 0.89–1.82)	1.20 ± 0.07	1.30 ± 0.14^a^	1.18 ± 0.08^b^	1.35 ± 0.17^a^ ^,^ ^c^	66.605	**<0.001**
Vascular risk scores (range 0–3)	0.72 ± 0.94	0.76 ± 0.90	0.81 ± 0.90	0.71 ± 0.87	0.145	0.933
0 (%)	35 (53.8)	30 (51.7)	29 (46.0)	25 (52.1)	4.395	0.884
1 (%)	18 (27.7)	14 (24.1)	20 (31.7)	14 (29.2)		
2 (%)	7 (10.8)	12 (20.7)	11 (17.5)	7 (14.6)		
3 (%)	5 (7.7)	2 (3.4)	3 (4.8)	2 (4.2)		
Neuropsychological measures						
MMSE (range 24–30)	28.74 ± 1.19	28.66 ± 1.19	27.83 ± 1.37^a^ ^,^ ^b^	27.17 ± 1.75^a^ ^,^ ^b^	35.753	**<0.001**
AVLT‐IR (range 6–31)	18.92 ± 4.06	18.62 ± 4.37	15.05 ± 4.26^a^ ^,^ ^b^	14.94 ± 2.96^a^ ^,^ ^b^	17.448	**<0.001**
AVLT‐LDR (range 0–12)	6.86 ± 1.71	6.79 ± 2.27	3.65 ± 1.89^a^ ^,^ ^b^	3.10 ± 2.13^a^ ^,^ ^b^	57.762	**<0.001**
BVMT‐R‐IR (range 0–36)	25.26 ± 5.64	21.91 ± 6.47	17.86 ± 7.67^a^	16.21 ± 8.16^a^ ^,^ ^b^	46.134	**<0.001**
BVMT‐R‐LDR (range 0–12)	10.54 ± 1.78	9.38 ± 2.53	8.37 ± 3.04^a^	7.00 ± 3.78^a^ ^,^ ^b^	36.673	**<0.001**
AFT (range 6–33)	18.68 ± 4.28	17.95 ± 3.67	13.11 ± 3.37^a^ ^,^ ^b^	13.25 ± 2.91^a^ ^,^ ^b^	39.528	**<0.001**
BNT (range 13–29)	24.05 ± 3.04	24.43 ± 3.21	22.7 ± 3.63^b^	21.52 ± 3.30^a^ ^,^ ^b^	8.711	**<0.001**
DST (range 3–18)	12.95 ± 1.70	12.79 ± 2.33	11.84 ± 2.47^a^	12.17 ± 2.01	3.629	**0.014**
STT A (range 22–110)	43.74 ± 12.80	43.50 ± 9.35	52.14 ± 18.16^a^ ^,^ ^b^	55.35 ± 19.74^a^ ^,^ ^b^	20.099	**<0.001**
STT B (range 46–240)	110.83 ± 22.42	113.22 ± 26.89	132.62 ± 38.27^a^ ^,^ ^b^	136.58 ± 39.02^a^ ^,^ ^b^	22.034	**<0.001**
JLO (range 0–30)	21.98 ± 4.62	20.64 ± 4.63	20.73 ± 4.75	19.65 ± 5.34	2.258	0.082
Imaging measure						
Mean cortical thickness (mm) (range 2.12–2.58)	2.31 ± 0.06	2.30 ± 0.06	2.28 ± 0.08	2.27 ± 0.07	2.725	0.045
Hippocampus volume (cm^3^) (range 4.01–8.26)	6.53 ± 0.69	6.61 ± 0.68	6.39 ± 0.74	6.33 ± 0.59	1.952	0.122
Relative hippocampus volume (range 3.24–5.73)	4.71 ± 0.44	4.60 ± 0.43	4.56 ± 0.49	4.41 ± 0.50^a^	3.840	**0.010**
Plasma biomarkers						
Aβ42 (pg/ml) (range 0.57–18.14)	10.91 ± 2.59	10.17 ± 2.98	10.34 ± 2.71	9.14 ± 4.29	5.126	0.163
Aβ40 (pg/ml) (range 8.92–407.83)	195.94 ± 38.01	206.17 ± 60.49	195.15 ± 54.27	191.62 ± 81.29	4.387	0.223
Aβ42/Aβ40 ratio (range 0.010–0.102)	0.056 ± 0.012	0.051 ± 0.011^a^	0.055 ± 0.012	0.050 ± 0.017^a^	9.975	**0.019**
p‐tau181 (pg/ml) (range 0.59–5.17)	1.67 ± 0.57	1.84 ± 0.80	1.93 ± 0.76	2.48 ± 1.16^a^ ^,^ ^b^ ^,^ ^c^	15.115	**0.002**
NfL (pg/ml) (range 4.19–42.69)	12.04 ± 4.26	14.90 ± 5.71^a^	13.99 ± 5.46^a^	18.21 ± 8.86^a^ ^,^ ^c^	18.410	**<0.001**
T‐tau (pg/ml) (range 0.21–6.69)	2.23 ± 0.87	2.33 ± 0.94	2.46 ± 1.20	2.45 ± 0.97	2.521	0.472

*Note:* Data are represented as mean ± standard deviation or number (percent). Bold, *p* < 0.05.

Abbreviations: AFT, Animal Verbal Fluency Test; AVLT, Auditory Verbal Learning Test; Aβ, amyloid beta; BNT, Boston Naming Test; BVMT‐R, Brief Visuospatial Memory Test‐Revised; DST, Digit Span test; IR, immediate recall; JLO, Judgment of Line Orientation; LDR, long delay recall; MMSE, Chinese Version of the Mini‐Mental State Examination; NC, normal cognition; Obj‐SCD, objectively defined subtle cognitive decline; STT, Shape Trail Test; SUVR, standardized uptake value ratio.

^a^ Compared with Aβ− NC group, *p* < 0.05;

^b^ compared with Aβ+ NC group, *p* < 0.05;

^c^ compared with Aβ− Obj‐SCD group, *p* < 0.05.

### 3.2 Performance of plasma biomarkers in diagnostic groups

**FIGURE 1:**
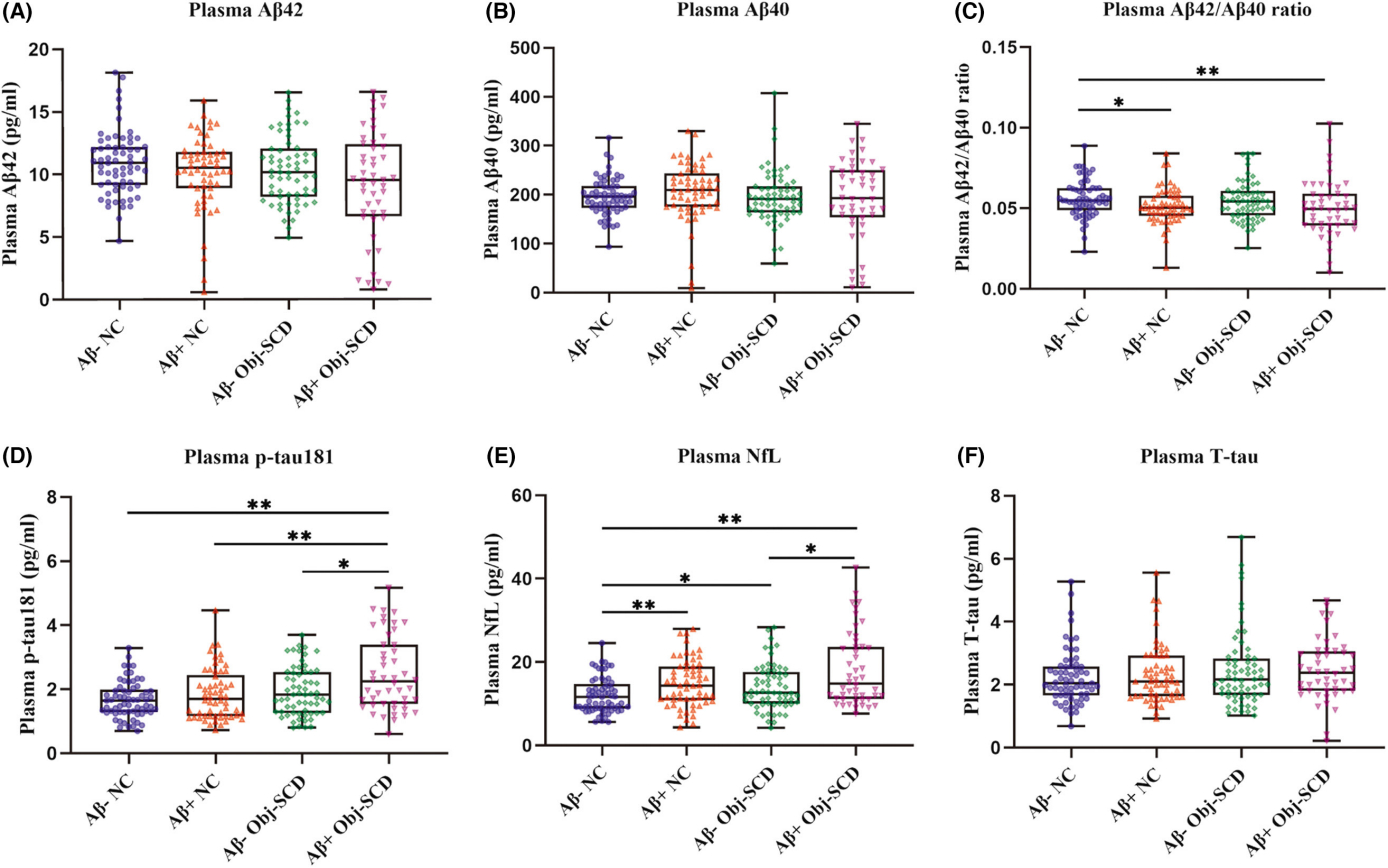
Performance of plasma biomarkers in diagnostic groups. Aβ, amyloid beta; NC, normal cognition; Obj‐SCD, objectively defined subtle cognitive decline. In box‐and‐whisker plots, the central horizontal bar shows the median, and the lower and upper boundaries show the 25th and 75th percentiles. *p < 0.05. **p < 0.01.

In plasma biomarkers, Aβ42/Aβ40 ratio was decreased in the Aβ+ NC and Aβ+ Obj‐SCD groups than Aβ− NC group. In contrast, plasma p‐tau181 levels were significantly increased in the Aβ+ Obj‐SCD group than the other three groups. Plasma NfL was elevated from the Aβ− NC group to the Aβ+ Obj‐SCD group and showed significant differences from Aβ− NC group. (Table 1, Figure 1).

We further analyzed the correlations between plasma biomarkers and demographic data. Plasma p‐tau181 and NfL were associated with age (*r* = 0.132–0.391, *p* < 0.043), but all plasma biomarkers were not associated with education level (*p* > 0.159). In addition, there was no significant difference between plasma biomarkers and sex, APOE ε4, and vascular risk scores.

To avoid confounding bias, we adjusted for age, sex, education, APOE ε4 status, and vascular risk scores. Plasma p‐tau181 was significantly higher in the Aβ+ Obj‐SCD group than in the two NC groups (*p* = 0.007–0.008). In addition, compared to Aβ− NC participants, plasma NfL was significantly increased in the Aβ+ Obj‐SCD group (*p* = 0.008). In comparison, other plasma biomarkers showed no significant difference between groups. The detailed information was listed in Table S1.

### 3.3 Associations between plasma biomarkers and brain amyloid burden

Associations between each plasma biomarker and brain amyloid burden are shown in Table 2. After adjustments for age, sex, education, APOE ε4 status, vascular risk scores and clinical diagnosis, logistic regression analysis found that plasma Aβ42 [OR (odds ratio) = 0.714, *p* = 0.017], Aβ42/Aβ40 ratio (OR = 0.717, *p* = 0.026), p‐tau181 (OR = 1.691, *p* = 0.002), and NfL (OR = 2.051, *p* < 0.001) were significant predictors for the brain amyloid positive. After adjusting for above covariates, independently linear regression showed that Aβ42/Aβ40 ratio (β = −0.134, *p* = 0.038), p‐tau181 (β = 0.274, *p* < 0.001), and NfL (β = 0.164, *p* = 0.020) was significantly correlated with SUVR. Both logistic and linear regression showed that p‐tau181 and NfL were the best predictors of Aβ deposition.

**TABLE 2:** Associations between individual plasma biomarkers and brain amyloid burden

Predictors	Amyloid PET status								
	Unadjusted			Adjusted^a^			Adjusted^b^		
	OR	95% CI	*p* Value	OR	95% CI	*p* Value	OR	95% CI	*p* Value
Aβ42	0.741	0.567, 0.967	**0.028**	0.729	0.555, 0.956	**0.022**	0.714	0.542, 0.941	**0.017**
Aβ40	1.072	0.827, 1.389	0.601	1.030	0.79, 1.344	0.826	1.022	0.782, 1334	0.876
Aβ42/Aβ40 ratio	0.702	0.528, 0.933	**0.015**	0.720	0.538, 0.964	**0.028**	0.717	0.534, 0.961	**0.026**
p‐tau181	1.602	1.178, 2.178	**0.003**	1.597	1.167, 2.186	**0.003**	1.691	1.216, 2.353	**0.002**
NfL	1.877	1.353, 2.605	**<0.001**	1.963	1.354, 2.846	**<0.001**	2.051	1.407, 2.990	**<0.001**
T‐Tau	1.108	0.847, 1.449	0.455	1.119	0.850, 1.473	0.422	1.135	0.860, 1.500	0.371

Predictors	Florbetapir PET SUVR								
	Unadjusted			Adjusted^a^			Adjusted^b^		
	β coefficient	95% CI	*p* Value	β coefficient	95% CI	*p* Value	β coefficient	95% CI	*p* Value
Aβ42	−0.123	−0.0141, −0.105	0.059	−0.119	−0.137, −0.101	0.062	−0.119	−0.137, −0.101	0.063
Aβ40	0.053	0.035, 0.071	0.424	0.027	0.009, 0.045	0.680	0.028	0.010,0.046	0.671
Aβ42/Aβ40 ratio	−0.163	−0.181, −0.145	**0.012**	−0.135	−0.153, −0.117	**0.038**	−0.134	−0.152, −0.116	**0.038**
p‐tau181	0.296	0.280, 0.312	**<0.001**	0.266	0.250, 0.282	**<0.001**	0.274	0.256, 0.292	**<0.001**
NfL	0.188	0.170, 0.206	**0.004**	0.161	0.143, 0.179	**0.020**	0.164	0.146, 0.182	**0.020**
T‐Tau	0.042	0.024, 0.060	0.521	0.077	0.059, 0.095	0.229	0.077	0.059, 0.095	0.233

*Note*: Results from binary logistic regression for the dichotomous outcome and linear regression for the continuous outcome. The regression models were independently performed for each plasma biomarker and adjusted for covariates. Bold, *p* < 0.05.

Abbreviations: PET, positron emission tomography; SUVR, standardized uptake value ratio; Aβ, amyloid beta; OR, odds ratio; 95% CI, confidence interval at 95%.

^a^ Adjusted for age, sex, education, APOE ε4 status, and vascular risk scores.

^b^ Adjusted for age, sex, education, APOE ε4 status, vascular risk scores, and clinical diagnosis.

### 3.4 Associations between plasma biomarkers and cognition domains

In all participants, Aβ42 was found to have weak correlation with visuospatial function (β = 0.143, *p* = 0.029). Higher p‐tau181 was widespread related to worse cognition in all cognition domains except attention (β = −0.337–0.232, *p* < 0.028). Elevated NfL was associated with the decline in global cognition, verbal episodic memory, visual episodic memory, and executive function (β = −0.336–0.244, *p* < 0.004). Plasma T‐tau was weakly related with language function (β = −0.140, *p* = 0.033).

Further sub‐group and adjustment analyses showed some different results. Associations between each plasma biomarker and cognition domain were examined using independent linear regression with adjustment for age, sex, education, APOE ε4 status, and vascular risk scores (Table 3). In Aβ− NC group, we found a negative correlation between p‐tau181 and visual episodic memory (β = −0.281, *p* = 0.031), and between NfL and verbal episodic memory (β = −0.285, *p* = 0.031); plasma T‐tau was correlated with attention (β = −0.291, *p* = 0.021) and visuospatial function (β = 0.269, *p* = 0.015). In Aβ+ NC individuals, higher p‐tau181 was associated with better executive function (β = −0.316, *p* = 0.022), while higher NfL and T‐tau were associated with worse MMSE (β = −0.395 to −0.329, *p* < 0.035). In Aβ− Obj‐SCD group, NfL had a negative correlation with verbal episodic memory (β = −0.338, *p* = 0.023), and T‐tau was negatively correlated with attention (β = −0.301, *p* = 0.019). In Aβ+ Obj‐SCD group, low Aβ42/Aβ40 ratio was related with poor verbal episodic memory (β = 0.286, *p* = 0.033); elevated p‐tau181 was moderately associated with worse performance in visual episodic memory (β = −0.522, *p* < 0.001), executive function (β = 0.518, *p* < 0.001), and visuospatial function (β = −0.404, *p* = 0.004).

**TABLE 3:** Associations between each plasma biomarker and cognition domain by groups

	Global cognition	Verbal episodic memory	Visual episodic memory	Language function	Attention	Executive function	Visuospatial function
Aβ− NC							
Aβ 42	−0.076	−0.207	−0.155	0.078	−0.021	−0.010	0.200
Aβ40	−0.062	−0.090	0.024	0.107	−0.100	−0.171	−0.016
Aβ42/Aβ40 ratio	−0.047	−0.136	−0.237	−0.045	0.065	0.148	0.183
p‐tau181	−0.172	0.128	−0.281*	−0.019	−0.162	−0.077	0.059
NfL	−0.162	−0.285*	−0.053	−0.119	−0.146	0.080	0.156
T‐tau	0.205	−0.001	−0.040	−0.115	−0.291*	−0.003	0.269*
Aβ+ NC							
Aβ 42	−0.189	−0.066	0.014	−0.188	−0.174	0.106	0.038
Aβ 40	−0.195	−0.050	−0.089	0.089	−0.264	0.112	−0.044
Aβ42/Aβ40 ratio	−0.026	−0.003	0.159	−0.041	0.156	0.024	0.118
p‐tau181	−0.111	−0.019	−0.016	0.044	0.181	−0.316*	0.117
NfL	−0.329*	−0.201	0.192	−0.116	0.232	0.058	−0.063
T‐Tau	−0.395**	−0.066	0.047	−0.063	0.075	−0.212	0.015
Aβ− Obj‐SCD							
Aβ42	−0.022	−0.072	0.035	−0.050	0.148	0.066	0.140
Aβ40	−0.058	−0.206	0.083	0.041	0.018	0.168	−0.046
Aβ42/Aβ40 ratio	0.097	0.148	−0.080	−0.048	0.093	−0.156	0.049
p‐tau181	0.170	−0.128	−0.126	−0.028	0.001	0.097	0.036
NfL	−0.078	−0.338*	−0.217	−0.002	−0.073	0.094	−0.044
T‐tau	0.122	0.092	−0.113	−0.060	−0.301*	−0.033	0.048
Aβ+ Obj‐SCD							
Aβ42	−0.001	0.067	0.007	0.096	−0.073	0.151	0.147
Aβ40	−0.110	−0.028	−0.041	0.151	−0.108	0.199	−0.031
Aβ42/Aβ40 ratio	0.043	0.286*	0.139	−0.096	0.062	−0.042	0.248
p‐tau181	−0.256	−0.099	−0.522**	−0.206	−0.037	0.518**	−0.404**
NfL	0.133	0.003	−0.042	0.121	0.136	0.167	−0.135
T‐tau	0.072	0.135	0.011	−0.156	0.021	0.065	0.116

*Note:* Data are represented as β and significant difference. Bold, *p* < 0.05. The regression models were independently performed for each plasma biomarker and adjusted for age, sex, education, APOE ε4 status, and vascular risk scores. *p < 0.05; **p < 0.01.

Abbreviations: Aβ, amyloid beta; NC, normal cognition; Obj‐SCD, objectively defined subtle cognitive decline.

### 3.5 Associations between plasma biomarkers and imaging measures

Among all participants, p‐tau181 was negatively correlated with relative hippocampus volume (β = −0.270, *p* < 0.001). NfL showed a weak and negative correlation with mean cortical thickness (β = −0.161, *p* = 0.014) and relative hippocampus volume (β = −0.232, *p* < 0.001).

We further adjusted for age, sex, education, APOE ε4 status, and vascular risk scores. Independent linear regression revealed that only Aβ42/Aβ40 ratio was positively associated with cortical thickness in Aβ− NC group (β = 0.279, *p* = 0.032). In addition, only high plasma p‐tau181 was significantly associated with reduced relative hippocampus volume in Aβ− Obj‐SCD group (β = −0.235, *p* = 0.034) and Aβ+ Obj‐SCD group (β = −0.330, *p* = 0.037).

### 3.6 Diagnostic performance of plasma biomarkers to identify Aβ+ Obj‐SCD

**FIGURE 2:**
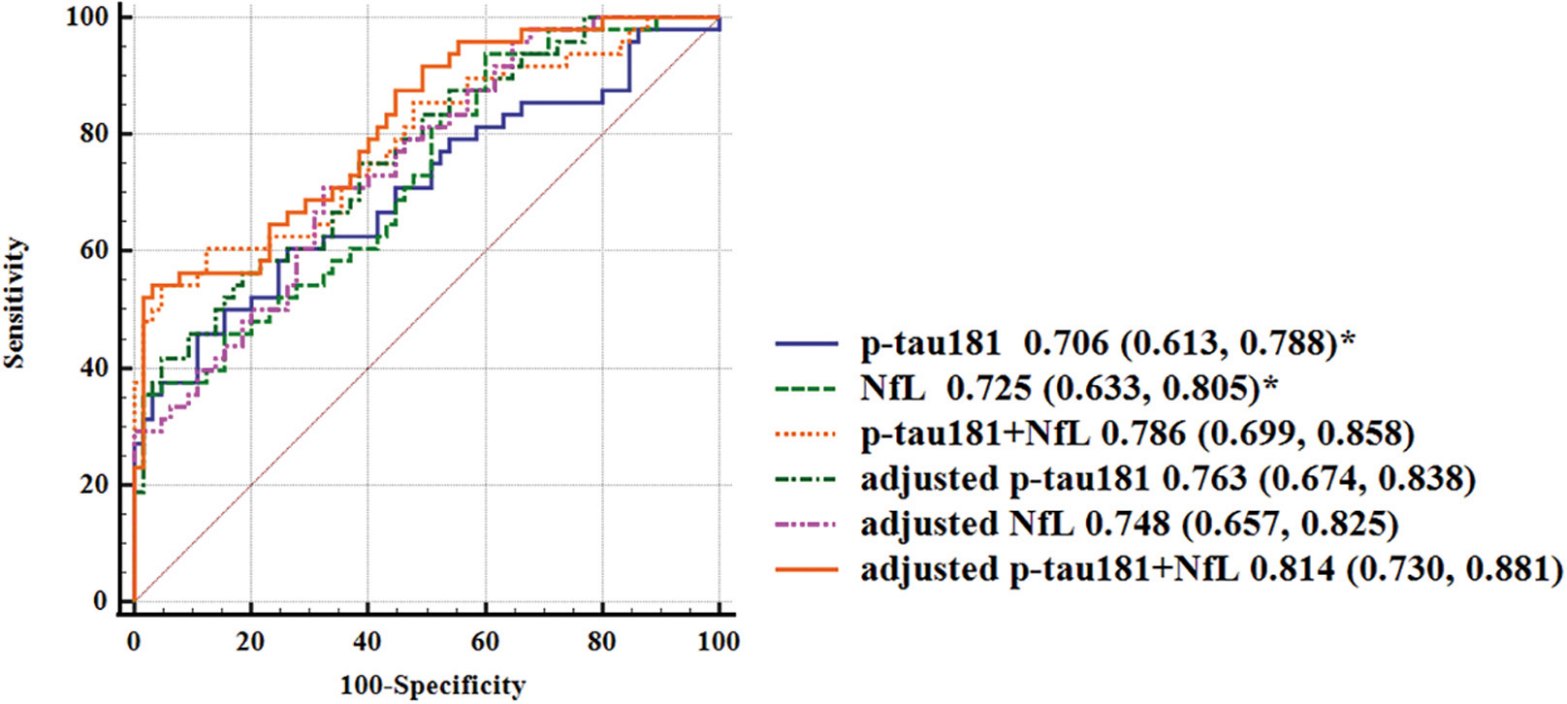
ROC analyses for distinguishing Aβ+ Obj‐SCD and Aβ‐ NC. ROC, receiver operating characteristic; Aβ, amyloid beta; NC, normal cognition; Obj‐SCD, objectively defined subtle cognitive decline; AUC, area under the curve; 95% CI, 95% confidence intervals. Adjusted for age, sex, education, APOE ε4 status, and vascular risk scores. *Compared with adjusted p‐tau181 + NfL, *p* < 0.05.

Given that p‐tau181 and NfL were significantly elevated in the Aβ+ Obj‐SCD group relative to Aβ− NC group, we next assessed their diagnostic efficacy between Aβ+ Obj‐SCD and Aβ− NC with or without adjustment. The biomarkers showed a moderate diagnostic ability, with the AUC ranging from 0.706 to 0.814 (Figure 2). After adjusting for age, sex, education, APOE ε4 status, and vascular risk scores, combining p‐tau181 and NfL showed the best diagnostic efficacy (AUC = 0.814) with a sensitivity of 64.58% and specificity of 76.52%. The best model did not differ from the adjusted model with p‐tau181 (AUC = 0.763) or NfL (AUC = 0.748), but was significantly superior to the single p‐tau181 (AUC = 0.706) and NfL (AUC = 0.725) model (*p* < 0.05).

## 4 DISCUSSION

The present study demonstrated that plasma biomarkers are differentially associated with brain amyloid, cognition, and neuroimaging measures. Among them, p‐tau181 and NfL were elevated in Aβ+ Obj‐SCD patients compared to NC and strongly correlated with brain amyloid and cognitive decline. Besides, p‐tau181 and NfL showed relatively good diagnostic capacity in identifying AD pathology Obj‐SCD from NC. Our findings further confirm the great potential of p‐tau181 and NfL for early AD screening in clinical trials.

In our study, the Aβ42 and Aβ42/Aβ40 ratio showed no difference between groups after adjustment. However, a low Aβ42/Aβ40 ratio was associated with amyloid load, poor verbal memory, and thin cortical thickness. Some studies found that Aβ42, Aβ40, and Aβ42/Aβ40 ratio progressively decreased with AD progression.^43^, ^44^ Lower Aβ42/Aβ40 ratio was found to be associated with increased cortical Aβ and tau in AD‐related regions.^45^ Study showed plasma Aβ42/Aβ40 had a high correspondence with amyloid PET status (AUC 0.88) and CSF p‐tau181/Aβ42 (AUC 0.85).^12^ Others found that low plasma amyloid at baseline was related to a steeper rate of subsequent cognitive decline in memory, attention, and executive function.^10^ Recent work showed contrary results that plasma Aβ40 and Aβ42 increased with the progression of AD, and the cortical thickness was negatively correlated with plasma Aβ40 and Aβ42.^46^ The predictive power of plasma Aβ42/Aβ40 for amyloid pathology varies widely.^9^, ^47^, ^48^ This inconsistent result may be due to different detection methods. Various assays for plasma analytes have widely varying performance.^49^, ^50^

Several studies have confirmed that plasma p‐tau181 is a diagnostic and prognostic biomarker for AD in cross‐sectional and longitudinal studies.^13^, ^51^ In our research, p‐tau181 was elevated in Aβ+ Obj‐SCD relative to NC. It was associated with Aβ deposition, visual episodic memory, executive and visuospatial function, and hippocampal volume, and these correlations were mainly in the AD pathology individuals. Our findings support previous studies that have been reported. A previous study suggested that plasma p‐tau181 showed a continuous escalating trend as the clinical dementia rating (CDR) scores increased from 0 (NC group) to 3 (severe AD). It mainly correlated with global cognition, memory, attention, and visuospatial function.^52^ A follow‐up visit found plasma p‐tau181 was associated with PET‐measured cerebral tau (AUC = 83.08%–93.11%) and Aβ (AUC = 76.14%–88.09%) pathologies, and 1‐year cognitive decline and hippocampal atrophy.^18^ Longitudinal changes of plasma p‐tau181 were associated with a prospective decrease in glucose metabolism, gray matter volume, and neurodegeneration. These associations were restricted to Aβ‐positive individuals.^15^, ^53^ In addition, plasma p‐tau181 was reported to show a stronger network association to AD than NfL and T‐tau.^54^ Another paradoxical finding in our study is that p‐tau181 had a positive effect on executive function in preclinical AD, yet a negative effect in the Aβ+ Obj‐SCD group. It was proven that p‐tau181 had an S‐shaped relationship with global cognition and multiple cognitive domains and thought that it only negatively affects cognitive function when a certain threshold is breached.^55^ The nonlinear relationship may be due to neuronal compensation and cognitive resilience in early AD to sustain executive function and cognitive flexibility.^56^ After extensive Aβ and tau accumulation, protective and compensatory mechanisms fail, leading to neurodegeneration and cognitive decline.^57^

NfL and T‐tau blood levels were considered biomarkers of axonal injury and neuronal damage.^58^ We found that plasma NfL was elevated in Aβ+ Obj‐SCD, and elevated NfL was associated with brain Aβ accumulation and worse verbal memory decline. Baseline and follow‐up studies demonstrated that high NfL levels were associated with increased biomarkers of neuronal injury in CSF, atrophy in temporal cortex thickness and hippocampal volume, hypometabolism, and worse global cognition and memory.^21^, ^47^, ^58^ In our cross‐sectional study, NfL was primarily associated with cognition in the Aβ‐negative group. However, longitudinal studies indicated that increased plasma NfL was related to AD‐related hypometabolism in individuals with positive amyloid pathology.^59^ Other analyses revealed that the rate of change in NfL predicted future PET tau pathology,^60^ disease progression, and brain neurodegeneration at the early presymptomatic stages of familial AD.^61^ Future longitudinal studies are required to explore the role of NfL and cognitive trajectories in the AD spectrum disorders.

In contrast, we found that T‐tau was unrelated to brain amyloid burden, whereas plasma T‐tau was associated with attention and visuospatial function in Aβ− group. Studies have confirmed that plasma tau partly reflects AD pathology, but there is a significant overlap between normal aging and AD, especially in patients without dementia.^24^ Recent research indicated higher levels of plasma T‐tau declines in global cognition, memory, attention, and visuospatial ability over a median follow‐up of 3.0 years.^25^ Thus, plasma NfL is superior to T‐tau as a neurodegeneration‐related biomarker in AD patients, which has been demonstrated in previous studies.^62^, ^63^ This further supports the idea that NfL and T‐tau reflect different aspects of neurodegeneration. The variable performance may also be related to the limitations of plasma T‐tau measurement methods, with a weaker correlation between plasma T‐tau and CSF T‐tau.^24^, ^64^ Notably, after adjusting for vascular factors in the correlation analysis, NfL and T‐tau remained linearly related to cognition. In addition, these correlations were predominantly present in the Aβ‐negative group, suggesting that NfL and T‐tau can affect cognitive decline independent of vascular factors and brain Aβ.^25^, ^65^ The correlation performance of each biomarker varies depending on clinical diagnosis and Aβ status, which may reflect different underlying mechanisms in different disease stages.

In the present study, single p‐tau181 and NfL achieved similar and moderate diagnostic efficacy in discriminating Aβ+ Obj‐SCD from Aβ− NC. After adjustment, the combination of plasma p‐tau181 and NfL could reach an AUC of 0.814, which was the first report on the diagnostic efficacy of blood biomarkers in identifying Obj‐SCD. Recent work found that blood p‐tau181 and NfL achieved diagnostic validity of 0.74 and 0.61, respectively, in predicting Aβ− versus Aβ+ participants.^66^ In a large cohort, plasma p‐tau181 showed excellent diagnostic power in distinguishing AD dementia from Aβ− young adults (AUC = 99.40%) and cognitively unimpaired older adults (AUC = 90.21–98.24%) and other neurodegenerative diseases (AUC = 81.90–92.13%).^18^ Another study revealed that higher plasma NfL levels accurately discriminated participants with AD dementia from both NC (AUC = 0.83) and MCI (AUC = 0.78).^67^ Both plasma p‐tau181 (AUC = 0.91) and NfL (AUC = 0.93) accurately distinguished pathology‐confirmed AD from NC.^68^ The variation in diagnostic value is due to different study samples and assays and the presence or absence of brain Aβ in the clinical diagnosis. Higher p‐tau181 and NfL levels were associated with a risk of clinical AD incidence within 9 years of diagnosis in a community‐based cohort study.^69^ Obj‐SCD is considered to be a particularly sensitive risk marker for future biomarker changes.^27^ When Obj‐SCD combining p‐tau181 and NfL can effectively identify AD‐related risks.

The present study has acknowledged limitations. First, given the cross‐sectional study design, we cannot track the trajectory of changes in biomarkers and the interrelationship with disease progression. Second, our sample was primarily drawn from the Shanghai community, which may limit the generalizability of our findings to other races. Large‐scale longitudinal studies are required to describe the change of plasma biomarkers from preclinical to symptomatic phases of the disease.

## 5 CONCLUSION

In conclusion, we found plasma p‐tau181 as diagnostic biomarkers related to AD and can help predict cognitive decline, even in predementia stages. Although NfL was non‐specific, it helped predict cognitive decline and offered complimentary diagnostic information. We further demonstrate the ability of these markers to identify AD‐related risks, supporting the potential use of plasma biomarkers in clinical trials. Future investigations on longitudinal changes of plasma biomarkers over time and a robust plasma biomarker framework are needed.

## CONFLICT OF INTEREST

The authors declare that they have no conflict of interest.

## Supporting information


Appendix S1

## Data Availability

The data during the current study are available from the corresponding author on reasonable request.
