# Supplementary material for: Associations of plasma phosphorylated tau181 and neurofilament light chain with brain amyloid burden and cognition in objectively defined subtle cognitive decline patients
Source: CNS Neurosci Ther. 2022 Sep 8;28(12):2195–205. doi: 10.1111/cns.13962 (PMC9627371; doi:10.1111/cns.13962)

Full unedited blot for Figure 1A

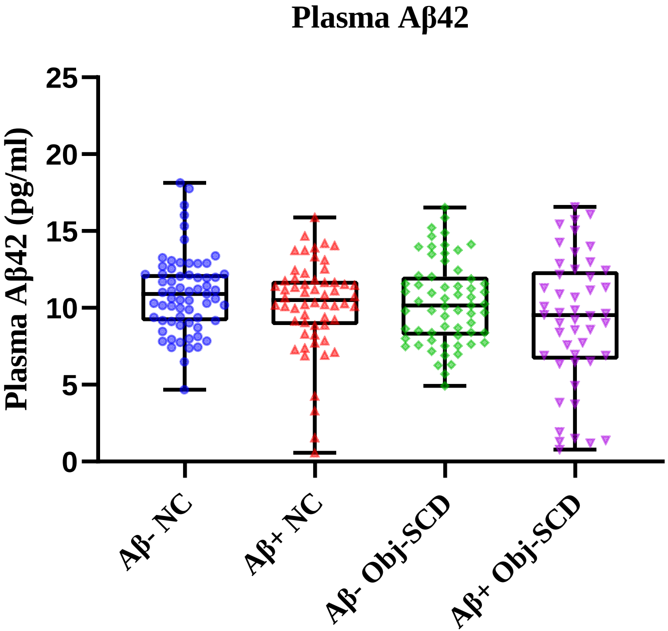

Full unedited blot for Figure 1B

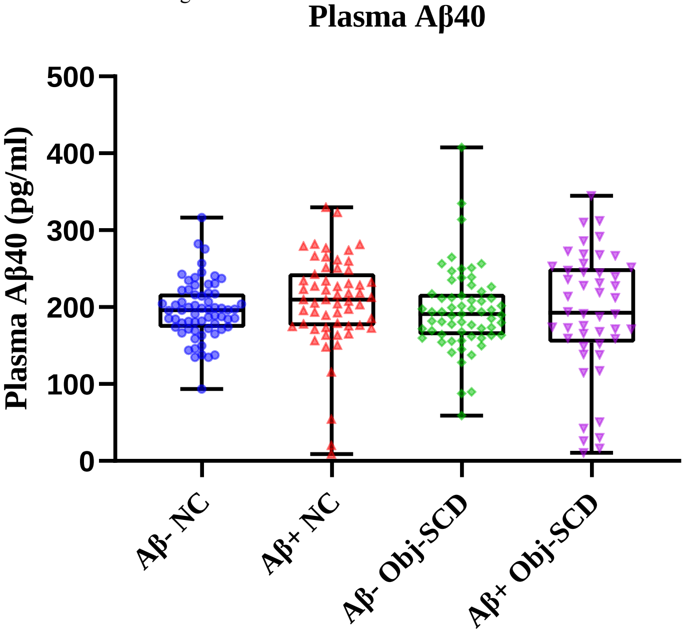

Full unedited blot for Figure 1C

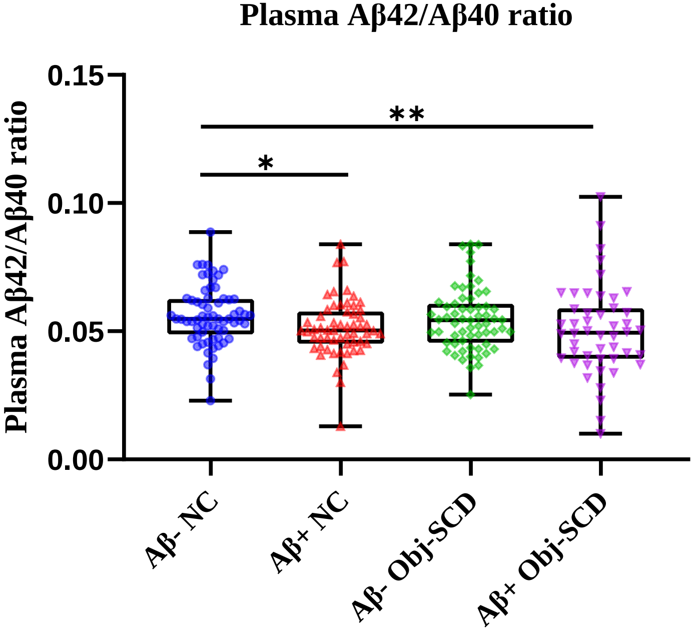

Full unedited blot for Figure 1D

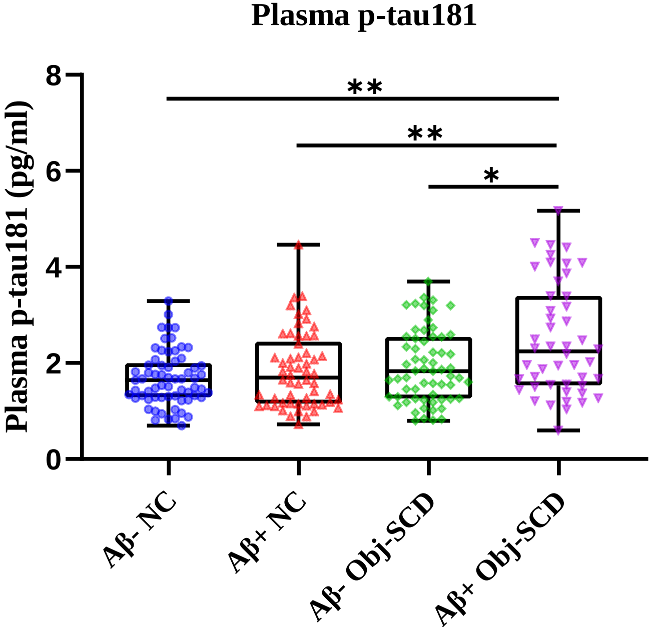

Full unedited blot for Figure 1E

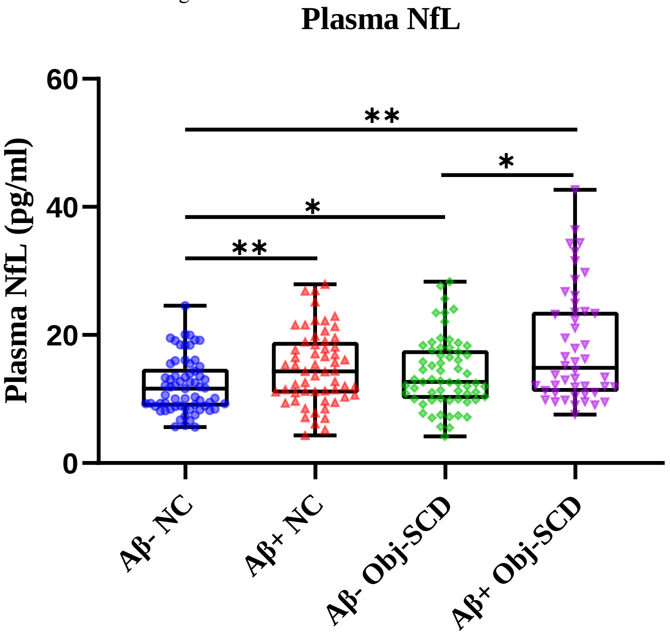

Full unedited blot for Figure 1F

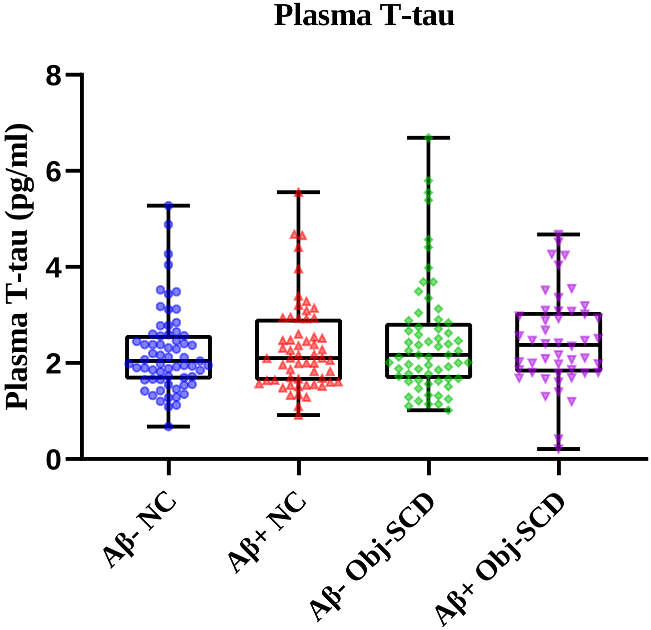

Full unedited blot for Figure 2

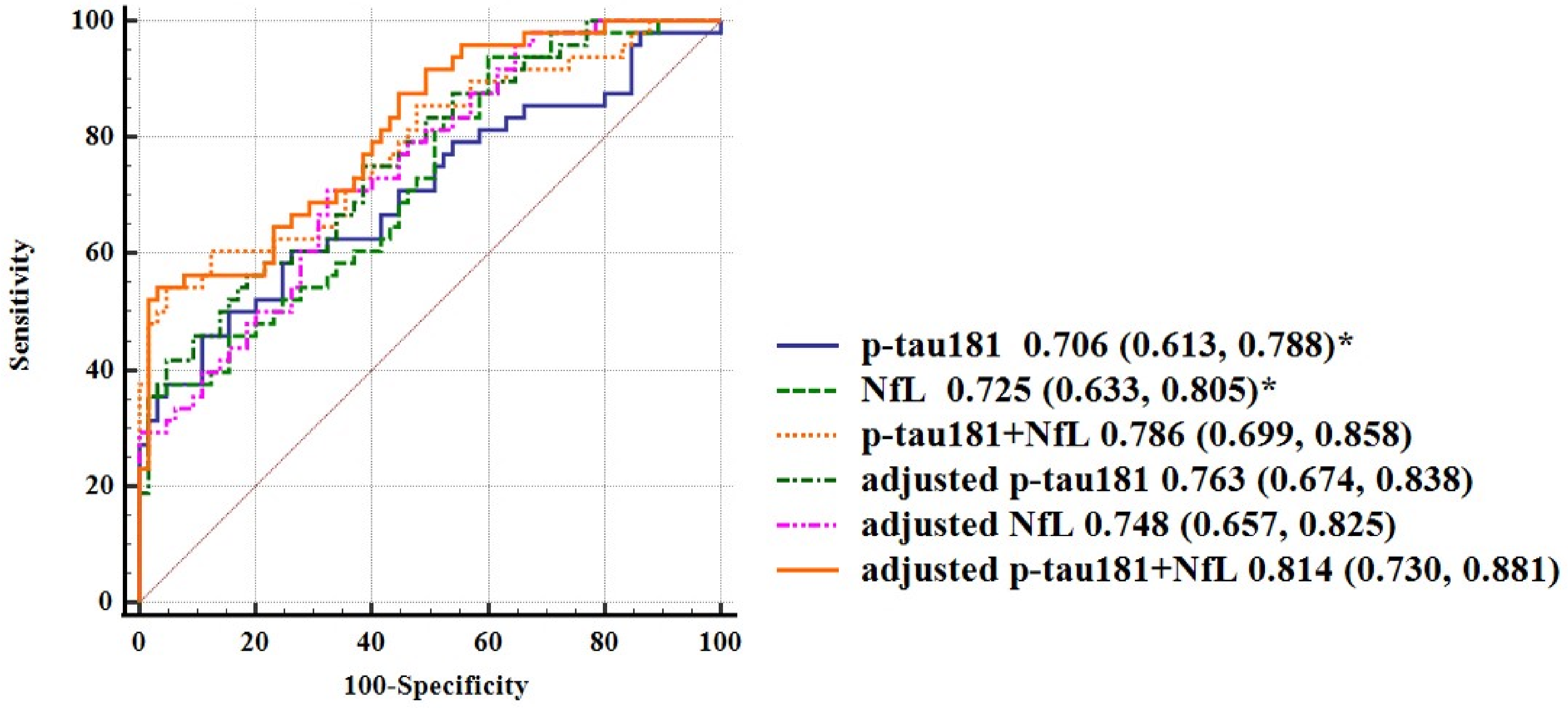

Supplement: Supplementary file 1 — Appendix S1 [file CNS-28-2195-s001.zip › CNS_13962_Supplemental Files.pdf]
