# Supplementary material for: Associations of plasma phosphorylated tau181 and neurofilament light chain with brain amyloid burden and cognition in objectively defined subtle cognitive decline patients
Source: CNS Neurosci Ther. 2022 Sep 8;28(12):2195–205. doi: 10.1111/cns.13962 (PMC9627371; doi:10.1111/cns.13962)
Supplement: Supplementary file 1 — Appendix S1 [file CNS-28-2195-s001.zip › CNS_13962_Table S1.pdf]

TABLE S1. Performance of plasma biomarkers in diagnostic groups

Plasma Biomarkers	Aβ- NC	Aβ+ NC	Aβ- Obj-SCD	Aβ+ Obj-SCD	F	p value
Aβ42 (pg/ml)	10.61 ± 0.58	10.41 ± 0.49	10.24 ± 0.55	9.63 ± 0.62	0.492	0.688
Aβ40 (pg/ml)	199.72 ± 10.76	215.45 ± 9.05	194.59 ± 10.25	209.42 ± 11.42	0.918	0.433
Aβ42/Aβ40 ratio	0.054 ± 0.003	0.050 ± 0.002	0.054 ± 0.002	0.048 ± 0.003	1.371	0.253
p-tau181 (pg/ml)	1.75 ± 0.18	1.84 ± 0.15	2.01 ± 0.17	2.63 ± 0.19 *†	4.539	**0.004**
NfL (pg/ml)	12.51 ± 1.18	14.84 ± 0.99	14.33 ± 1.12	18.17 ± 1.25 *	3.688	**0.013**
T-tau (pg/ml)	2.14 ± 0.27	2.21 ± 0.23	2.70 ± 0.26	2.43 ± 0.29	0.947	0.419

Note: Data are represented as mean ± standard deviation. Adjusted for age, sex, education, APOE ε4 status, and vascular risk scores. Bold, $p < 0.05$. * compared with Aβ- NC group, $p < 0.05$; † compared with Aβ+ NC group, $p < 0.05$.

Abbreviations: Aβ, amyloid beta; NC, normal cognition; Obj-SCD, objectively defined subtle cognitive decline.
